# The neonatal lung microbiome: a dynamic determinant of respiratory health, disease, and novel therapeutics

**DOI:** 10.3389/fped.2026.1770578

**Published:** 2026-03-16

**Authors:** Wanwei Zheng, Yan Liang, Jiayao Li

**Affiliations:** 1 Department of Pediatrics, People’s Hospital of Ming Shan District Ya'an, Ya'an City, Sichuan, China; 2 School of Clinical Medicine & The First Affiliated Hospital of Chengdu Medical College, Chengdu, Sichuan, China

**Keywords:** dysbiosis, lung microbiome, microbiome therapeutics, neonatal respiratory disorders, probiotics, short-chain fatty acids

## Abstract

The neonatal lung, once considered sterile, is now recognized to harbor a dynamic and complex microbiome that plays a critical role in respiratory health and disease. This review synthesizes current evidence on the composition, development, and functional impact of the lung microbiome in neonates, with a focus on its involvement in key respiratory disorders such as bronchopulmonary dysplasia, respiratory syncytial virus infection, neonatal acute respiratory distress syndrome, cystic fibrosis, and asthma predisposition. We place particular emphasis on the bidirectional communication along the gut-lung axis as a central mechanism, wherein intestinal microbiota and their metabolites modulate pulmonary immunity and inflammation. Emerging multi-omics studies that integrate microbial data with host metabolomic and immune profiles are highlighted for their role in deciphering disease-specific dysbiotic signatures and mechanistic pathways. Critically, this review advances the discussion beyond association by evaluating the translational potential of the microbiome as both a diagnostic biomarker and a therapeutic target. We provide a critical appraisal of innovative microbiome-targeted strategies—including probiotics, postbiotics, phage therapy, and bacterial lysates—and discuss the unique challenges and future directions for translating these approaches into safe, effective clinical interventions for vulnerable neonates. By bridging foundational science with clinical implications, this work aims to inform the development of novel, ecology-informed therapeutics to prevent and mitigate neonatal respiratory diseases.

## 1 Introduction

Neonatal respiratory disorders remain a leading cause of morbidity and mortality worldwide, posing a significant challenge to global health systems (1). Among these, bronchopulmonary dysplasia (BPD), respiratory syncytial virus (RSV) infection, neonatal acute respiratory distress syndrome (NARDS), and early manifestations of conditions like cystic fibrosis (CF) and asthma disproportionately affect infants, particularly those born prematurely (2). The pathogenesis of these diseases is multifactorial, involving genetic predisposition, environmental exposures, immature immune responses, and aberrant lung development (3). For decades, therapeutic strategies have primarily focused on supportive care, anti-inflammatory agents, and antimicrobials, with varying degrees of success and often accompanied by side effects.

Historically, the lower respiratory tract, particularly in neonates, was widely believed to be a sterile environment (4). This “sterile lung” paradigm was largely sustained by the limitations of conventional culture-based microbiological techniques, which failed to detect the sparse, fastidious, or uncultivable microorganisms residing in the lungs (5–7). The advent and widespread application of culture-independent molecular technologies, most notably 16S ribosomal RNA gene sequencing and metagenomic shotgun sequencing, has fundamentally overturned this view (8, 9). A growing body of foundational studies has now unequivocally demonstrated the presence of a diverse, albeit low-biomass, community of bacteria, viruses, and fungi in the lungs from the earliest stages of life, including in both term and preterm infants (10–13). This microbial assemblage, now recognized as the “lung microbiome,” is understood not as a mere collection of passive inhabitants but as an active and integral component of the pulmonary ecosystem.

This discovery signifies a profound paradigm shift in our understanding of respiratory health and disease, positioning the lung microbiome as a critical determinant (14, 15). Far from being passive, this microbial community actively participates in shaping pulmonary immunity, maintaining epithelial barrier integrity, and modulating inflammatory responses. Furthermore, the lung does not function in isolation (16). The concept of the gut-lung axis has gained substantial experimental support, delineating a complex, bidirectional communication network (17–19). Through this axis, the intestinal microbiota and its metabolic products [e.g., short-chain fatty acids (SCFAs), tryptophan metabolites] exert systemic immunomodulatory effects on the lung, influencing susceptibility to and severity of respiratory conditions; conversely, pulmonary inflammation can feedback to alter gut microbial ecology (20–22).

Emerging evidence links specific patterns of lung dysbiosis to clinical outcomes. For instance, a decreased diversity and a shift towards dominance by *Proteobacteria* (e.g., *Haemophilus*, *Klebsiella*) or *Firmicutes* (e.g., *Streptococcus*) have been associated with the development and progression of BPD, more severe RSV infection, and NARDS (23–25). Conversely, the presence of certain commensals like *Lactobacillus*, *Dolosigranulum*, and *Corynebacterium* appears to be associated with milder disease or protective effects (26–28). These associations suggest that the neonatal lung microbiome may serve not only as a biomarker for disease risk and progression but also as a novel therapeutic target.

This review aims not only to synthesize the current understanding of the role of the lung microbiome in the pathogenesis of major neonatal respiratory disorders but also to critically evaluate its emerging translational potential. We will explore the dynamic development of the early-life pulmonary microbiota, its interaction with the host immune system via the gut-lung axis, and its specific alterations in conditions such as BPD, RSV, NARDS, and CF. Furthermore, we will provide a forward-looking appraisal of microbiome-targeted biotherapeutics, discussing their mechanisms, current evidence, and the specific challenges involved in their application to neonates. By bridging insights from multi-omics, clinical, and preclinical studies, this review seeks to elucidate how harnessing the lung microbiome could pave the way for innovative, personalized strategies to prevent, mitigate, or treat debilitating respiratory diseases in this vulnerable neonatal population.

## 2 Literature search strategy

This narrative review aimed to synthesize current evidence on the neonatal lung microbiome and its role in respiratory health and disease. To ensure a comprehensive and reproducible overview of the field, a structured literature search was conducted. The following electronic databases were systematically queried from their inception until April 2025: PubMed/MEDLINE, Web of Science Core Collection, and Embase.

The search strategy employed a combination of controlled vocabulary (e.g., MeSH terms in PubMed) and free-text keywords related to three core concepts (1): Population: neonate, newborn, preterm, infant, “early life”. (2) Intervention/Exposure: “lung microbiome”, “respiratory microbiome”, “airway microbiota”, “gut-lung axis”. (3) Outcome/Disorder: “bronchopulmonary dysplasia”, “BPD”, “respiratory syncytial virus”, “RSV”, “neonatal acute respiratory distress syndrome”, “NARDS”, “cystic fibrosis”, “asthma”, “dysbiosis”, “probiotics”, “short-chain fatty acids”. Boolean operators (AND, OR) were used to combine these concepts. The search string for PubMed is provided below: (neonate OR newborn OR preterm OR infant) AND (“lung microbiome” OR “respiratory microbiome” OR “airway microbiota”) AND (bronchopulmonary dysplasia OR BPD OR “respiratory syncytial virus” OR RSV OR “acute respiratory distress syndrome” OR cystic fibrosis OR asthma). The reference lists of retrieved relevant reviews and primary articles were also manually screened to identify additional pertinent studies.

Study Selection and Inclusion Criteria: Records were initially screened based on title and abstract. Full-text articles of potentially eligible studies were then assessed. Inclusion criteria were: (1) original research articles (clinical, translational, or preclinical) or authoritative reviews; (2) focus on the neonatal or early infancy period; (3) investigation of the lung/respiratory microbiome and/or the gut-lung axis; and (4) relevance to respiratory health, disease pathogenesis, or therapeutic interventions. Articles not in English, those focusing exclusively on adults or older children without neonatal data, and studies limited to the upper respiratory tract without linkage to lung health were excluded.

**Figure 1:**
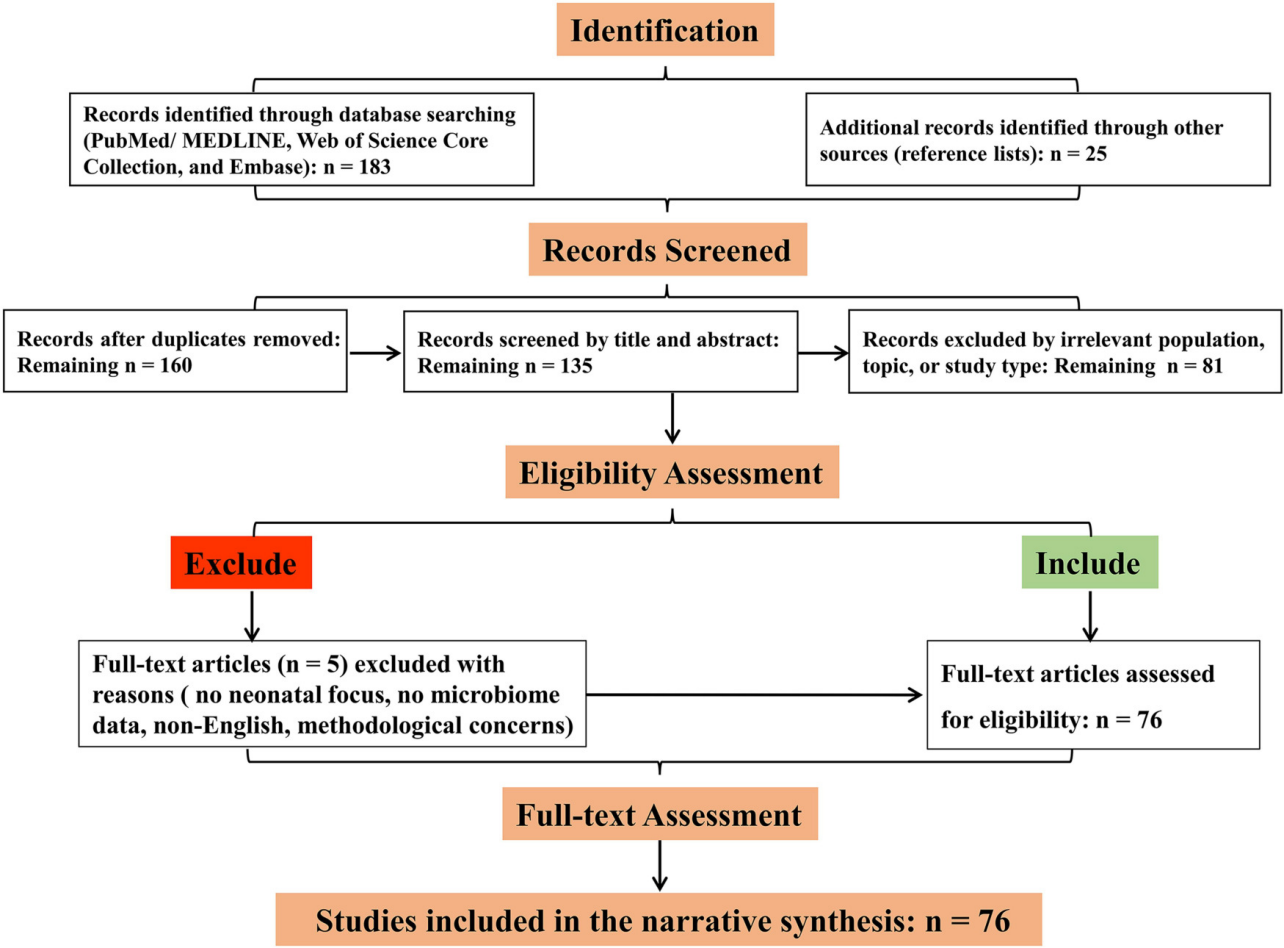
Flow diagram of literature identification and selection.

Data Synthesis: Given the narrative nature of this review, qualitative synthesis was not performed. Identified literature was organized thematically to address the review's objectives: elucidating the development of the neonatal lung microbiome, its role in specific diseases, the mechanisms of the gut-lung axis, and emerging therapeutic strategies. The selection and interpretation of studies were guided by the goal of providing a balanced, state-of-the-art perspective on the field. A flow diagram illustrating the study selection process is presented in Figure 1.

## 3 Development and determinants of the neonatal lung microbiome

**Figure 2:**
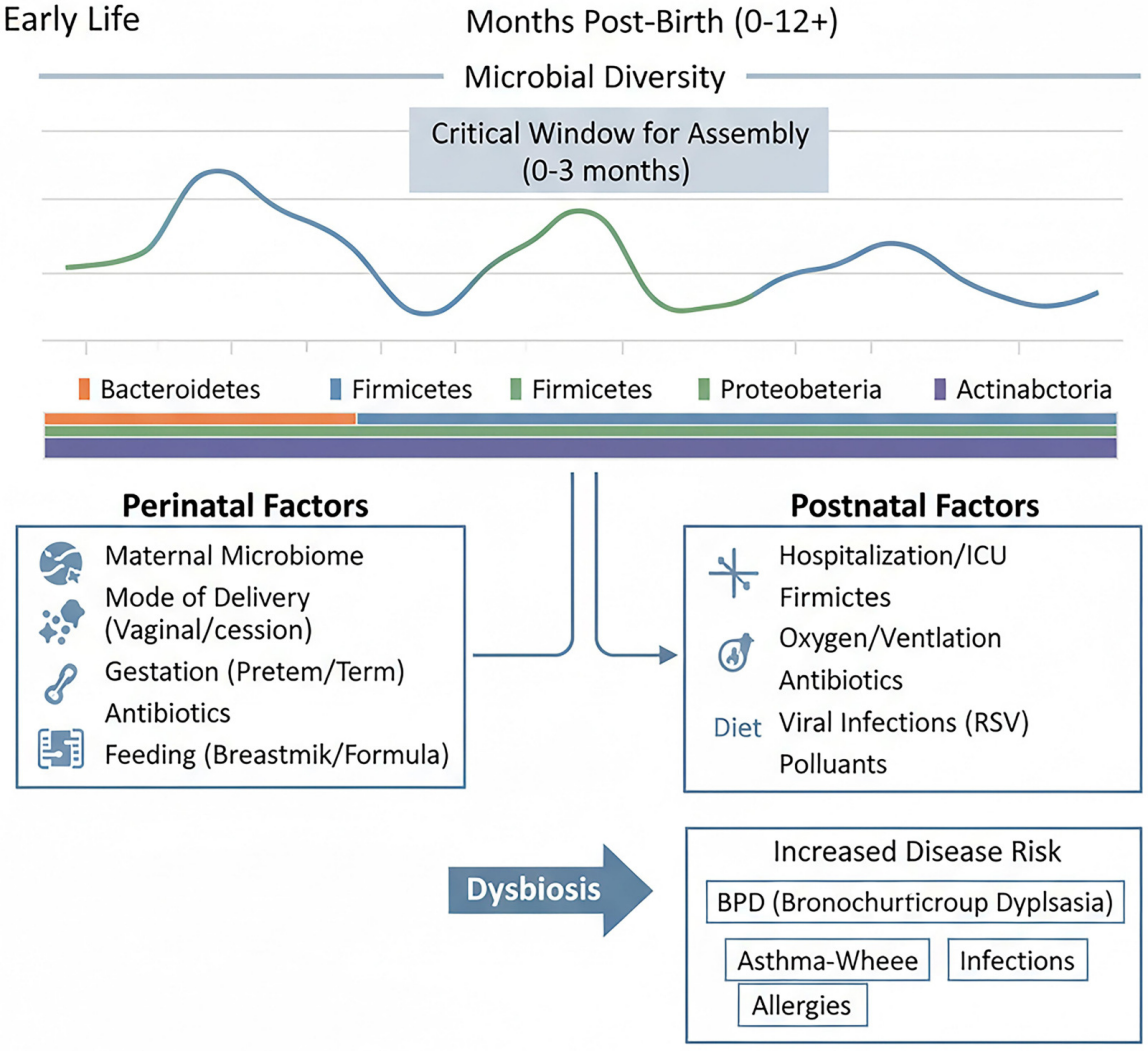
Developmental trajectory and determinants of the neonatal lung microbiome. The timeline depicts the increase in microbial diversity during early life, with a critical window for assembly in the first months. Colored bars indicate shifts in major bacterial phyla. Key perinatal and postnatal factors influencing microbiome composition and driving dysbiosis are shown, with links to increased disease risk. BPD, bronchopulmonary dysplasia; RSV, respiratory syncytial virus.

Contrary to the long-held belief of sterility, contemporary research utilizing high-throughput sequencing confirms that the lower respiratory tract is colonized by microbial communities from the earliest moments of life (29, 30). In both term and preterm infants, an established and diverse airway microbiome is detectable at birth, suggesting potential fetal or intrauterine acquisition (29, 31). This early community is dynamic and undergoes significant maturation during the first weeks and months of life. In murine models, lung microbial diversity increases with age, stabilizing into a more resilient community in adulthood (30). A critical window for this assembly appears to exist within the first 2–4 months of life in humans, a period characterized by significant compositional shifts and heightened susceptibility to pathogenic colonization (32) (Figure 2).

The initial composition and subsequent trajectory of the neonatal lung microbiome are shaped by a complex interplay of intrinsic and extrinsic factors. Gestational age is a primary determinant, with preterm birth dramatically altering the exposure timeline and microenvironment. Preterm infants often face immediate exposure to nosocomial bacteria facilitated by endotracheal intubation and mechanical ventilation (33). Perinatal factors such as chorioamnionitis and mode of delivery influence the initial microbial inoculum (29). Postnatal exposures are profoundly impactful: antibiotic use depletes commensal bacteria and promotes dysbiosis (34, 35); oxygen therapy (hyperoxia) confers a selective growth advantage for oxygen-tolerant pathogens like *Staphylococcus aureus* and disrupts both lung and gut microbial communities, exacerbating injury (35, 36); and nutrition, particularly human milk feeding, appears to support a healthier microbiome, potentially through prebiotic oligosaccharides and immunomodulatory factors (34) (Figure 2).

In addition to these perinatal and therapeutic factors, early-life viral respiratory infections represent a significant environmental force that reshapes the developing lung microbiome. Infections with common pathogens such as respiratory syncytial virus (RSV) have been shown to induce profound and specific alterations in the respiratory microbial community. These changes are characterized by a reduction in overall bacterial diversity and a shift in composition, often marked by an increased relative abundance of potential pathobionts such as *Haemophilus*, *Streptococcus*, and *Moraxella* in the nasopharynx and airways (37, 38). This virus-associated dysbiosis is not merely a transient effect; it can disrupt the stable assembly of the early-life microbiome, compromise local immune homeostasis, and may create a niche conducive to secondary bacterial complications. The interplay between specific viral infections, particularly RSV, and the resultant microbial dysbiosis is a critical determinant of disease severity and convalescence, a relationship explored in detail later in this review (Figure 2).

Furthermore, the neonatal lung microbiome is intricately linked to microbial communities at other body sites, particularly the gut. Cross-niche network analyses reveal that strong microbial connections between the oral cavity, nasopharynx, and gut established in the first week of life are associated with susceptibility to subsequent respiratory tract infections (39). This underscores the systemic nature of early microbial colonization and its central role in programming respiratory health (Figure 2).

Implications and Future Directions: Understanding these determinants underscores the potential for modifiable factors (e.g., prudent antibiotic use, human milk promotion) to positively shape the neonatal lung microbiome. Future research should employ longitudinal, multi-omics designs to decipher how early-life exposures program microbial trajectories and subsequent disease risk.

## 4 The Gut-lung axis: A critical pathway for immune programming

The gut-lung axis represents a pivotal bidirectional communication network where the intestinal microbiota and its metabolites regulate pulmonary immunity and inflammation (31, 40, 41). This axis is especially crucial in early life when both systemic and mucosal immune systems are undergoing education and maturation.

**Figure 3:**
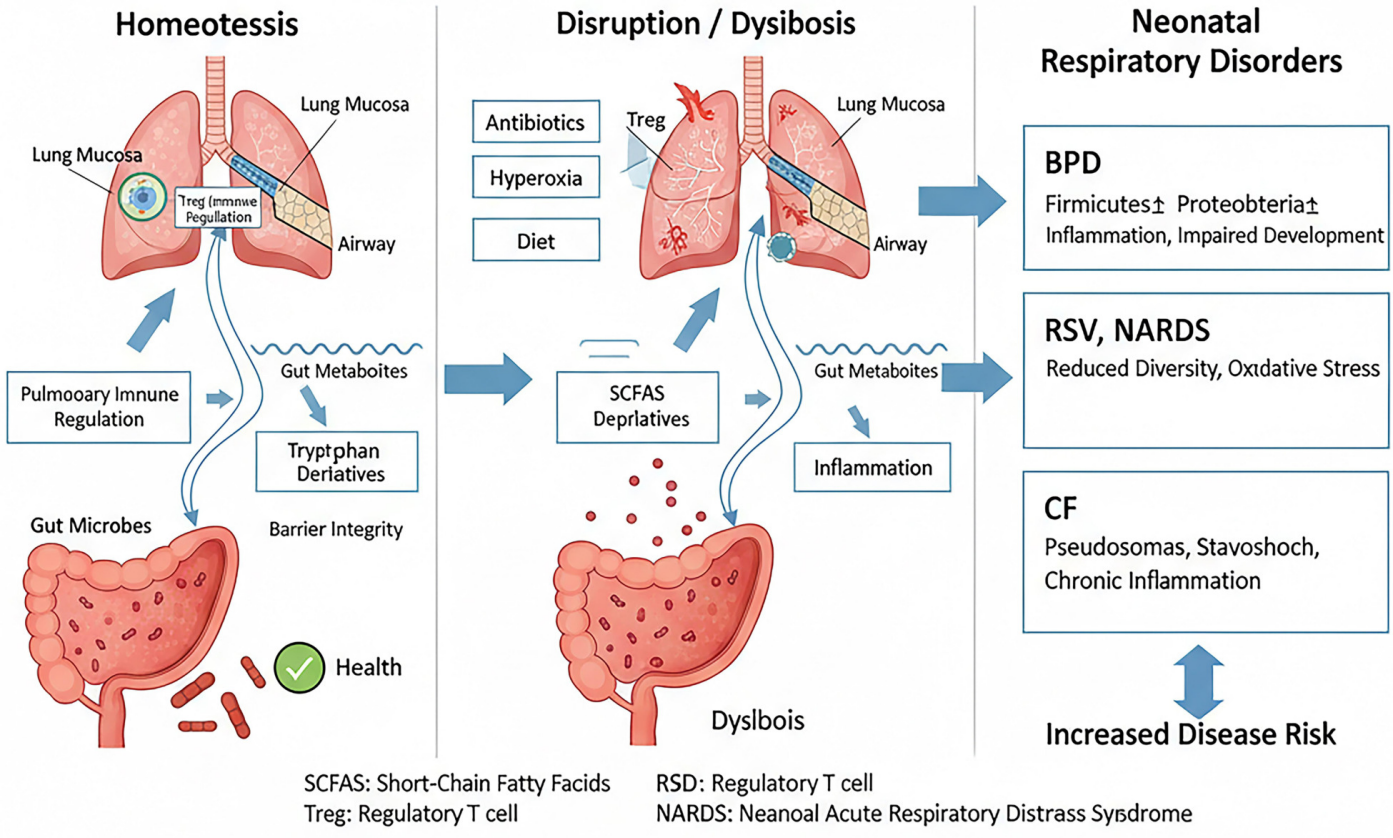
The gut-lung axis in health and disease. In homeostasis, gut-derived microbial metabolites (SCFAs, tryptophan derivatives) promote pulmonary immune regulation and barrier integrity. Disruption by antibiotics, hyperoxia, or diet leads to dysbiosis. This dysbiosis drives pathophysiology in major neonatal respiratory disorders, characterized by distinct microbial and immune profiles. SCFAs, short-chain fatty acids; Treg, regulatory T cell; AhR, aryl hydrocarbon receptor; BPD, bronchopulmonary dysplasia; RSV, respiratory syncytial virus; NARDS, neonatal acute respiratory distress syndrome; CF, cystic fibrosis.

Key microbial metabolites serve as signaling molecules along this axis. SCFAs, such as butyrate, acetate, and propionate, produced by gut bacteria from dietary fiber fermentation, have demonstrated systemic anti-inflammatory and immunomodulatory effects. They can enhance epithelial barrier function, promote the differentiation of regulatory T cells, and modulate macrophage function, thereby influencing the severity of conditions like BPD and asthma (41, 42). Tryptophan metabolites, including indole derivatives and kynurenine pathway products, are another crucial class of immune modulators. Alterations in lung and plasma tryptophan metabolite levels are closely linked to NARDS and BPD, and are correlated with specific microbial shifts (43–45) (Figure 3).

Disruption of this metabolic communication, often due to antibiotic-induced dysbiosis, can impair respiratory immunity. For instance, dysbiosis reduces intestinal production of inosine, a metabolite essential for programming effective influenza-specific CD8+ T cell responses in infants via the NFIL3-TCF1 axis (46). Similarly, a maternal high-fat diet can induce neonatal dysbiosis and low-grade systemic inflammation, predisposing offspring to severe viral lower respiratory tract infections and subsequent asthma through neutrophil-mediated IL-6 trans-signaling (47). Crucially, the communication along the gut-lung axis is bidirectional. While the gut influences the lung, signals originating from the lung also exert a powerful effect on gut homeostasis. Pulmonary inflammation, a hallmark of conditions like hyperoxia-induced lung injury or severe infection, can increase systemic and intestinal levels of pro-inflammatory cytokines, alter gut barrier permeability, and subsequently reshape the composition and function of the gut microbiota (48). This is exemplified by early-life respiratory viral infections such as RSV, which have been shown to induce not only acute airway dysbiosis but also long-term alterations in the gut microbiome. These persistent gut microbial shifts can contribute to an increased risk of post-viral wheezing and allergic asthma, highlighting how a primary lung insult can program distant organ susceptibility via the gut (49). Furthermore, lung-derived mediators—including inflammatory cells, cytokines, and potentially even microbial components or metabolites translocated from the inflamed lung—may directly or indirectly modulate the intestinal microenvironment (50). This feedback loop underscores the gut-lung axis as a truly integrated system, wherein perturbations at either site can propagate and amplify dysfunction at the other, with significant implications for neonatal disease pathogenesis and persistence. These findings illustrate how perturbations at one end of the gut-lung axis can manifest as disease at the other (Figure 3).

Implications and Future Directions: The gut-lung axis presents a powerful lever for intervention. Future studies need to delineate the precise molecular signals and immune circuits involved in this cross-talk, and investigate whether modulating gut microbiota can consistently and safely improve pulmonary outcomes in neonates.

## 5 Dysbiosis in specific neonatal respiratory disorders

### 5.1 BPD

BPD, a chronic lung disease of prematurity, is strongly associated with a distinct and evolving airway dysbiosis. Preterm infants who develop BPD exhibit reduced bacterial diversity in their airways from birth, a pattern that persists over time compared to preterm controls without BPD (33, 51). Longitudinal studies show greater microbial community turnover in infants progressing to severe BPD (33). Common dysbiotic signatures include a predominance of *Proteobacteria* (e.g., *Escherichia*, *Klebsiella*) and *Firmicutes* (e.g., *Streptococcus*), coupled with a decrease in beneficial commensals like *Bacteroidetes*, *Lactobacillus*, and *Prevotella* (35, 43, 51). This dysbiosis is not merely an epiphenomenon; a Gammaproteobacteria-predominant microbiome is linked to increased endotoxin levels and can attenuate the protective Nrf2-mediated antioxidant response in the lung, exacerbating hyperoxia-induced injury (35). Conversely, colonization with *Lactobacillus* species or supplementation with *L. reuteri* and its tryptophan metabolite can ameliorate BPD-like injury in animal models by activating protective pathways such as IL-22/STAT3 (35, 44) (Table 1, Figure 3).

**Table 1:** Summary of Key microbial dysbiosis features in Major neonatal respiratory disorders.

Respiratory disorder	Core dysbiotic features	Key taxa associated with disease (↑ Increase, ↓ Decrease)	Clinical & pathogenic correlates	Refs
Bronchopulmonary Dysplasia	Reduced *α*-diversity, increased community turnover over time.	↑ *Proteobacteria* (e.g., *Escherichia*, *Klebsiella*), *Streptococcus*, *Ureaplasma*; ↓ *Bacteroidetes*, *Lactobacillus*, *Prevotella*.	Associated with prolonged oxygen/antibiotic use, worse lung injury; linked to attenuated Nrf2 antioxidant response.	(35, 43, 51)
Severe respiratory syncytial virus Infection	Distinct microbiota at time of infection vs. healthy controls.	↑ *Haemophilus*, *Streptococcus*, *Moraxella*; ↓ *Dolosigranulum*, *Corynebacterium*.	Predicts disease severity, prolonged symptoms, and post-viral wheezing/asthma risk.	(52, 53)
Neonatal ARDS	Altered lung-gut microbiota and tryptophan metabolism.	↑ *Proteobacteria*, *Bacteroidota*; ↓ *Firmicutes*, *Streptococcus*, *Rothia*, lung *Lactobacillus*.	Changes in lung microbiota and tryptophan metabolites show diagnostic potential for disease grading.	(43) (57)
Early Cystic Fibrosis	Decreased diversity with age, unstable microbial network.	Early pathogen colonization (e.g., *S. aureus*, *H. influenzae*); network fragmentation.	Associated with increased airway inflammation; early viral infections exacerbate bacterial inflammation.	(59, 60) (62, 63)

α-diversity, alpha diversity (within-sample microbial diversity); Nrf2, nuclear factor erythroid 2-related factor 2 (antioxidant response pathway). ↑ indicates increased relative abundance; ↓ indicates decreased relative abundance. Italicized bacterial names denote genus or species level. References correspond to studies cited in the main text.

### 5.2 RSV infection

The respiratory microbiome at the time of RSV infection is a key determinant of disease severity and convalescence. Infants with severe RSV lower respiratory tract infection harbor nasopharyngeal microbiota enriched with *Haemophilus*, *Streptococcus*, and *Moraxella* (52, 53). In contrast, higher abundances of *Dolosigranulum* and *Corynebacterium* are associated with milder disease (52). The gut microbiome also plays a role: RSV infection can induce long-term alterations in the gut microbiota, which contribute to an increased risk of post-viral wheezing and allergic asthma (54). Therapeutically, probiotic mixtures (e.g., containing *Lactobacillus rhamnosus GG*) have shown promise in murine models by modulating the gut-lung axis, increasing SCFA levels, restoring lung microbiota, and boosting alveolar macrophage-derived IFN-*β* responses to protect against RSV (55) (Table 1, Figure 3).

### 5.3 NARDS and pneumonia

NARDS is characterized by significant perturbations in both lung and gut microbiota, as well as in tryptophan metabolism (43, 56). The lungs of NARDS neonates show increased *Proteobacteria* and *Bacteroidota* and decreased *Firmicutes* and *Streptococcus* (43). These microbial changes are closely correlated with alterations in lung tryptophan metabolites, offering potential diagnostic biomarkers (43). In neonatal pneumonia, high-throughput sequencing reveals a complex microbiota with key pneumonia-associated genera including *Streptococcus*, *Rothia*, and *Corynebacterium* (57). Integrating such microbial signatures with clinical data could enable more precise diagnosis and targeted antimicrobial therapy (Table 1, Figure 3).

### 5.4 CF and asthma predisposition

In CF, even in the absence of classic pathogens, the lower airway microbial network is less stable and more prone to fragmentation from early infancy (58). Early viral infections, particularly with *rhinovirus*, are common and associated with increased neutrophilic inflammation and bacterial pathogen recovery, potentially initiating a cycle of inflammation and infection (59, 60). Longitudinal studies show that microbial diversity decreases with age and pathogen dominance is linked to greater inflammation (61).

Early-life lung microbial colonization is also critical for asthma susceptibility. Appropriate bacterial stimuli in the neonatal period are essential for inducing regulatory T cells via PD-L1, promoting tolerance to aeroallergens (62). Conversely, dysbiosis or the absence of specific protective bacterial strains (e.g., certain *Lactobacillus* species) during this critical window can lead to sustained susceptibility to allergic airway inflammation (62, 63). Early-life RSV infection further compounds this risk by altering both lung and gut microbiomes, creating a milieu conducive to allergic predisposition (54) (Table 1, Figure 3).

Implications and Future Directions: The gut-lung axis presents a powerful lever for intervention. Future studies need to delineate the precise molecular signals and immune circuits involved in this cross-talk, and investigate whether modulating gut microbiota can consistently and safely improve pulmonary outcomes in neonates.

## 6 Microbiome-Targeted therapeutic strategies

The recognition of the neonatal lung and gut microbiota as active participants in respiratory health and disease has catalyzed the development of novel therapeutic interventions. These strategies aim not merely to eradicate pathogens, but to restore ecological balance (eubiosis), modulate host immunity, and promote lung resilience. Current approaches can be categorized into several complementary paradigms, ranging from direct microbial supplementation to the targeted application of microbial-derived effector molecules.

### 6.1 Direct microbial supplementation: probiotics and live biotherapeutics

The most direct strategy involves administering beneficial live microorganisms. While traditionally focused on gut health, accumulating evidence now supports the concept of respiratory probiotics or live biotherapeutics for lung diseases (64, 65). Specific strains, such as *Lactobacillus reuteri* and *Lactobacillus rhamnosus* GG, have demonstrated efficacy in preclinical models of neonatal respiratory disorders (66, 67). Their mechanisms are multifaceted: they can directly compete with pathogens for niche occupancy, produce antimicrobial compounds, and critically, modulate the host immune system. In hyperoxia-induced BPD models, intranasal administration of *L. reuteri* significantly ameliorated lung injury, reduced pro-inflammatory cytokines (IL-1β, IL-6, TNF-α), and enhanced expression of lung maturation markers (SP-C, AQP5) (66, 68). This protective effect was mechanistically linked to the activation of the IL-22/STAT3 signaling pathway (44). Similarly, a probiotic mixture administered orally protected neonatal mice from RSV infection by reversing gut and lung dysbiosis, increasing systemic levels of microbiota-derived SCFAs, and boosting antiviral type I interferon (IFN-β) production by alveolar macrophages (55). These studies underscore that probiotic effects can be mediated both locally in the lung and remotely via the gut-lung axis (Tables 2, 3).

**Table 2:** Key Microbiota-derived metabolites and their roles in neonatal lung health and disease.

Metabolite class	Primary microbial source	Key immunomodulatory mechanisms	Demonstrated role in neonatal respiratory disorders
Short-Chain Fatty Acids (e.g., Butyrate, Acetate)	Gut commensals (*Firmicutes*, *Bacteroidetes*)	Activate GPCRs (e.g., GPR41, GPR43); inhibit HDACs; promote Treg differentiation; enhance epithelial barrier.	Butyrate ameliorates asthma-like inflammation; acetate mediates probiotic protection against RSV via macrophage IFN-*β*.
Tryptophan Metabolites (e.g., Indole-3-aldehyde, IPA)	*Lactobacillus*, *Bifidobacterium* etc.	Activate Aryl Hydrocarbon Receptor (AhR); modulate IL-22 production.	I3Ald mimics *L. reuteri* in protecting against hyperoxic lung injury (BPD model). Altered levels correlate with NARDS severity.
Nucleosides (e.g., Inosine)	Gut bacteria	Serves as substrate/modulator for immune cells; regulates NFIL3-dependent T cell epigenetic programming.	Supplementation rescues dysbiosis-impaired CD8^+^ T cell immunity against influenza in neonates.

GPCRs, G protein-coupled receptors; HDACs, histone deacetylases; Treg, regulatory T cells; Ahr, aryl hydrocarbon receptor; I3Ald, indole-3-aldehyde; IPA, indole-3-propionic acid; IFN-β, interferon-beta; RSV, respiratory syncytial virus; BPD, bronchopulmonary dysplasia; NARDS, neonatal acute respiratory distress syndrome. All metabolites listed are derived from microbial metabolism.

**Table 3:** Overview of microbiome-targeted therapeutic strategies for neonatal respiratory disorders.

Therapeutic strategy	Definition & examples	Proposed mechanism of action	Current Stage & key findings	Key supporting references
Probiotics/Live Biotherapeutics	Administration of live beneficial bacteria (e.g., *L. reuteri*, *L. rhamnosus GG*, probiotic mixtures).	Competitive exclusion of pathogens; production of antimicrobials; immunomodulation (e.g., IL-22/STAT3, IFN-β induction).	Effective in preclinical models of BPD, RSV, hyperoxia. Clinical efficacy in neonates under active investigation.	(66–68)
Postbiotics	Administration of inactivated microbes or, more commonly, their bioactive metabolites (e.g., SCFAs, I3Ald, inosine).	Direct receptor-mediated signaling (GPCRs, AhR); epigenetic regulation; restoring specific deficient immune functions.	Metabolite supplementation (butyrate, inosine) rescues disease phenotypes in animal models, offering a precise pharmacological approach.	(44, 46, 69)
Bacteriophage Therapy	Use of specific viruses to infect and lyse pathogenic bacteria.	Highly specific bactericidal activity against target strains.	Compassionate use in drug-resistant *M. abscessus* infections shows safety and potential efficacy; requires personalization.	(70)
Immunomodulatory Bacterial Lysates	Oral administration of standardized extracts from inactivated bacteria (e.g., OM-85).	“Training” innate immunity via mucosal dendritic cells; enhancing barrier defense.	Large RCTs (e.g., PROTEA) ongoing to evaluate efficacy in preventing infections/wheeze in preterm infants.	(71)
Supportive & Adjunctive Strategies	Antimicrobial stewardship, human milk promotion, mitigating dysbiotic effects of oxygen/antibiotics.	Preserving commensal diversity and function; providing prebiotics (HMOs) and beneficial microbes.	Foundational for all microbiome health; evidence links human milk to reduced BPD risk.	(72–74)

BPD, bronchopulmonary dysplasia; RSV, respiratory syncytial virus; GPCRs, G protein-coupled receptors; Ahr, aryl hydrocarbon receptor; SCFAs, short-chain fatty acids; I3Ald, indole-3-aldehyde; HMOs, human milk oligosaccharides; RCTs, randomized controlled trials. Italicized bacterial names refer to specific strains. “Current stage” refers to evidence from preclinical models or clinical studies as referenced in the text.

### 6.2 Harnessing microbial metabolites: the post biotic approach

An alternative to administering live microorganisms is the use of postbiotics—defined as preparations of inactivated microbial cells or, more focally, their bioactive metabolic products. This strategy addresses challenges related to probiotic survival and engraftment in a dysbiotic or critically ill host by offering a more precise and pharmacologically controllable intervention. The therapeutic potential lies in directly supplementing key microbial-derived signaling molecules that are essential for immune regulation and barrier function. Among these, SCFAs—such as butyrate, acetate, and propionate, produced by gut bacterial fermentation of dietary fiber—act as potent immunomodulators. They exert systemic effects through mechanisms including G protein-coupled receptor activation and histone deacetylase inhibition, leading to enhanced regulatory T cell function and dampened neutrophilic inflammation. In models of asthma exacerbated by early-life stress, butyrate supplementation mitigated airway inflammation and restored epithelial barrier integrity by upregulating tight junction proteins like Occludin and E-cadherin (69). Another critical class comprises tryptophan metabolites. For instance, indole-3-aldehyde (3-IAld), produced by bacteria such as *Lactobacillus*, can activate the aryl hydrocarbon receptor and the protective IL-22/STAT3 pathway, demonstrating efficacy comparable to its parental live strain in ameliorating hyperoxia-induced lung injury in BPD models (44). Furthermore, nucleosides like inosine play a specific role in immune programming: depletion of intestinal inosine due to antibiotic-induced dysbiosis was shown to impair neonatal CD8^+^ T cell responses to influenza, and direct inosine supplementation rescued this defect by restoring NFIL3-dependent epigenetic regulation of T cell factor expression (46). Collectively, these findings underscore that targeted replenishment of defined microbial metabolites can correct specific immune or metabolic deficiencies arising from dysbiosis, providing a foundational rationale for developing next-generation, pharmacology-inspired respiratory therapeutics based on bacterial molecular signals (Tables 2, 3).

### 6.3 Precision antimicrobials: bacteriophage therapy

For infections caused by multidrug-resistant bacteria, such as *Mycobacterium abscessus* in cystic fibrosis, phage therapy offers a highly specific alternative. Phages are viruses that infect and lyse specific bacterial strains. The compassionate use of personalized phage cocktails (selected based on the susceptibility of the patient's own bacterial isolate) in patients with drug-resistant mycobacterial disease has shown promise, with favorable clinical or microbiological responses observed in over half of treated patients and no serious adverse events attributed to the therapy (70). The main challenges are the limited repertoire of therapeutically useful phages for many pathogens and the potential development of neutralizing antibodies, which may impact efficacy upon intravenous administration (Tables 2, 3).

### 6.4 Immune training with bacterial lysates

This strategy employs immunomodulatory preparations of inactivated bacterial components (lysates) to “educate” the innate immune system, enhancing its readiness to respond to pathogens without causing disease. The oral bacterial lysate OM-85, derived from common respiratory bacteria, is a prime example. Large randomized controlled trials like the PROTEA study are currently evaluating whether early-life administration of OM-85 can reduce the incidence of lower respiratory tract infections and wheezing in moderate-to-late preterm infants (71). The proposed mechanism involves priming mucosal dendritic cells and enhancing innate immune barriers, potentially shifting the immune milieu towards a more balanced, less inflammatory state upon subsequent pathogen encounter (Table 3).

### 6.5 Foundational and adjunctive supportive strategies

In parallel with the development of direct microbiome-targeted therapies, optimizing routine clinical care to protect and nurture a healthy neonatal microbiome represents a critical and often overlooked therapeutic foundation. These supportive strategies aim to prevent iatrogenic dysbiosis and create a physiological environment conducive to both host resilience and the efficacy of more specific interventions. Paramount among these is antimicrobial stewardship—judiciously limiting the duration and spectrum of antibiotic exposure to minimize collateral damage to commensal communities, thereby preserving microbial diversity and metabolic function. Concurrently, the promotion of human milk feeding serves as a powerful, multi-faceted microbiome-supportive intervention. Beyond nutrition, human milk provides human milk oligosaccharides that function as selective prebiotics, immunoglobulins with pathogen-neutralizing capacity, and a direct inoculum of beneficial bacteria, collectively fostering a healthier microbial ecosystem in the gut and, indirectly, the lung (72). Furthermore, as unavoidable interventions such as oxygen therapy are known to disrupt microbial homeostasis, there is a growing imperative to develop adjunctive strategies to mitigate their dysbiotic effects (73, 74). This could include the investigational use of antioxidants or specific metabolites to protect commensal niches during hyperoxia, or the refinement of ventilation practices to reduce microbial perturbation. Ultimately, these foundational measures are integral to a holistic therapeutic framework; by maintaining a more stable and resilient microbiome, they enhance the host's inherent defenses and create a more receptive environment for the success of targeted probiotics, postbiotics, or other advanced biotherapeutics (Table 3).

Implications and Future Directions: While preclinical data are promising, the path to clinic requires overcoming formulation, delivery, and safety challenges specific to neonates. Next steps include optimizing strain and metabolite selection, developing neonatal-appropriate delivery systems, and initiating rigorously designed pilot clinical trials that incorporate mechanistic endpoints.

## 7 Discussion

This review synthesizes the rapidly evolving evidence establishing the neonatal lung microbiome not as a passive bystander, but as a fundamental determinant of respiratory health and a key contributor to the pathogenesis of major respiratory disorders. The paradigm has shifted from a sterile lung model to recognizing a dynamic, low-biomass ecosystem that is seeded early in life and undergoes critical maturation during a vulnerable developmental window (29, 30, 32). Its composition and stability are exquisitely sensitive to perinatal and postnatal factors, including gestational age, mode of delivery, antibiotic exposure, oxygen therapy, and nutrition (34–36, 51). Crucially, the lung does not function in isolation; it is embedded within the systemic framework of the gut-lung axis, wherein intestinal microbial communities and their metabolites (e.g., SCFAs, tryptophan derivatives, inosine) exert profound immunomodulatory effects on pulmonary immunity (40, 41, 46). Dysbiosis—characterized by diminished diversity, loss of beneficial commensals, and overgrowth of potentially pathogenic taxa—emerges as a common pathological signature across diverse conditions such as BPD, severe RSV infection, NARDS, and early CF (35, 43, 51, 52). This association is increasingly supported by mechanistic studies in animal models, demonstrating that microbial dysbiosis can directly exacerbate inflammation, impair antioxidant defenses, and disrupt alveolar development (35, 36, 44).

The translational promise of this field lies in the development of microbiome-targeted biotherapeutics. Strategies range from direct microbial supplementation (probiotics) and the application of their bioactive products (postbiotics) to precision antimicrobials (phage therapy) and immune training with bacterial lysates (42, 44, 55, 70, 71). These approaches represent a move beyond broad-spectrum suppression towards ecological restoration and targeted modulation. Notably, interventions like specific *Lactobacillus* strains or metabolite supplements (e.g., butyrate, inosine) have shown efficacy not only in correcting dysbiosis but also in rectifying specific downstream immune defects, such as impaired CD8+ T cell responses or deficient IL-22/STAT3 signaling (44, 46). This underscores a shift towards a more mechanistic, “pathway-aware” therapeutic paradigm.

However, significant challenges must be navigated to realize this potential. First, establishing causality remains complex. While strong associations between dysbiosis and disease severity are evident, disentangling whether microbial changes are drivers of pathology, consequences of the disease state or its treatment, or merely epiphenomena requires sophisticated longitudinal studies and germ-free or gnotobiotic animal models (35, 54). Second, methodological standardization is urgently needed. Variations in sample collection (e.g., tracheal aspirate vs. bronchoalveolar lavage), DNA extraction protocols, sequencing platforms, and bioinformatic analyses hinder cross-study comparisons and the identification of universal biomarkers (75). The low biomass of lung samples also heightens susceptibility to contamination, demanding rigorous controls. Third, the personalization of therapy will be crucial. Given the high inter-individual variability in microbiome composition and the finding that phage efficacy is strain-specific, future interventions likely need to be tailored based on individual microbial and metabolomic profiles (32, 70).

Translating insights from the neonatal lung microbiome into clinical practice faces unique hurdles, some of which are magnified compared to the more established field of gut microbiome research. While gastrointestinal microbiome science has grappled with and begun to address issues of standardization, multi-omics integration, and personalized microbial therapeutics over the past two decades, respiratory microbiome research must accelerate through this learning curve. Key methodological challenges are more acute in the lung: the extremely low microbial biomass amplifies contamination risks, and sample acquisition (via tracheal aspirate, bronchoalveolar lavage) is more invasive and heterogeneous than stool collection, demanding even more rigorous controls and consortium-driven standardized protocols to enable robust cross-study comparisons. Therapeutic development encounters specific obstacles not as prominent in gut-focused interventions: ensuring safe and effective delivery of live biotherapeutics or metabolites to the lower airways, guaranteeing their engraftment in a dysbiotic or inflamed niche, and navigating the heightened safety imperatives for critically ill neonates. To bridge the gap from promising animal models to clinical trials, the field can draw lessons from successful gut-lung axis interventions. For instance, the design and outcomes of neonatal probiotic trials for preventing necrotizing enterocolitis or late-onset sepsis offer a template for evaluating safety, dosing, and strain selection in vulnerable populations. A concerted, multi-disciplinary effort is required—combining rigorous preclinical models that mimic neonatal pathophysiology, innovative drug delivery systems, and early-phase clinical studies that incorporate deep microbial and immune phenotyping—to build the evidence base necessary for targeted, safe, and effective microbiome-based respiratory therapeutics.

Looking ahead, the field of neonatal lung microbiome research is poised to move from descriptive associations to causative mechanisms and, ultimately, to personalized medicine. This transition will be fueled by several key avenues of investigation. First, deep multi-omics integration—simultaneously profiling the microbiome, host transcriptome, epigenome, metabolome, and proteome from serial samples—will be essential to construct causal network models of disease pathogenesis and identify master regulatory pathways amenable to intervention (45, 76). Second, leveraging artificial intelligence and machine learning on these complex datasets holds promise for identifying robust predictive biomarkers of disease susceptibility or therapeutic response, paving the way for early risk stratification (71). Third, the concept of personalized microbial therapeutics will likely mature. This may involve autologous probiotic approaches, engineered phage cocktails tailored to an individual's pathogen, or metabolite supplementation regimens guided by the patient's specific metabolic deficiencies. Finally, fostering interdisciplinary collaboration among neonatologists, microbiologists, immunologists, bioengineers, and computational biologists is critical to innovate solutions for the unique challenges of sample acquisition, mechanistic modeling, and therapeutic delivery in this vulnerable population. By embracing these perspectives, research can transform the neonatal lung microbiome from a fascinating ecological discovery into a cornerstone of next-generation respiratory care, aiming to restore microbial homeostasis and improve long-term pulmonary resilience.

In conclusion, the neonatal lung microbiome represents a pivotal interface between the environment, microbial ecology, and host immunity, playing a central role in the pathogenesis of respiratory disorders. While dysbiosis is a common hallmark of disease, it also unveils a rich new landscape for therapeutic intervention. By advancing our mechanistic understanding, standardizing research practices, and conducting rigorous clinical trials, the field can progress from correlation to causation and from promise to practice. Harnessing the microbiome holds the potential to transform neonatal respiratory care, moving towards strategies that promote resilience, restore balance, and ultimately improve long-term pulmonary outcomes for vulnerable infants.
